# Antibacterial Effects and Mode of Action of Selected Essential Oils Components against *Escherichia coli* and *Staphylococcus aureus*

**DOI:** 10.1155/2015/795435

**Published:** 2015-06-28

**Authors:** Julio Cesar Lopez-Romero, Humberto González-Ríos, Anabela Borges, Manuel Simões

**Affiliations:** ^1^Research Center for Food and Development, CIAD, A.C., 83000 Hermosillo, SON, Mexico; ^2^LEPABE, Department of Chemical Engineering, Faculty of Engineering, University of Porto, 4200-465 Porto, Portugal; ^3^CIQUP, Department of Chemistry and Biochemistry, Faculty of Sciences, University of Porto, Rua do Campo Alegre s/n, 4169-007 Porto, Portugal; ^4^Veterinary and Animal Science Research Center (CECAV), Department of Veterinary Science, University of Trás-os-Montes e Alto Douro, 5000-801 Vila Real, Portugal

## Abstract

Bacterial resistance has been increasingly reported worldwide and is one of the major causes of failure in the treatment of infectious diseases. Natural-based products, including plant secondary metabolites (phytochemicals), may be used to surpass or reduce this problem. The objective of this study was to determine the antibacterial effect and mode of action of selected essential oils (EOs) components: carveol, carvone, citronellol, and citronellal, against *Escherichia coli* and *Staphylococcus aureus*. The minimum inhibitory concentration (MIC) and minimum bactericidal concentration (MBC) were assessed for the selected EOs components. Moreover, physicochemical bacterial surface characterization, bacterial surface charge, membrane integrity, and *K* ^+^ leakage assays were carried out to investigate the antimicrobial mode of action of EOs components. Citronellol was the most effective molecule against both pathogens, followed by citronellal, carveol, and carvone. Changes in the hydrophobicity, surface charge, and membrane integrity with the subsequent *K* ^+^ leakage from *E. coli* and *S. aureus* were observed after exposure to EOs. This study demonstrates that the selected EOs have significant antimicrobial activity against the bacteria tested, acting on the cell surface and causing the disruption of the bacterial membrane. Moreover, these molecules are interesting alternatives to conventional antimicrobials for the control of microbial infections.

## 1. Introduction

Antimicrobial resistance is one of the most serious public health threats that results mostly from the selective pressure exerted by antibiotic use and abuse [1, 2]. During the last decades, rapid evolution and spread of resistance among clinically important bacterial species have been observed. Due to this growing increase of resistance, many antimicrobial agents are losing their efficacy [3–5]. Consequently, the therapeutic options for the treatment of infections have become limited or even unavailable. According to the World Health Organization (WHO) infectious diseases are the second cause of death around the world [6]. It is also estimated that antimicrobial resistance causes more than 2,049,442 illnesses and 23,000 deaths per year in the United States and these cases are increasing every year [7]. Therefore, it is necessary to develop new alternative compounds to decrease the problem of the microbial resistance.

Plants produce an enormous array of functional relevant secondary metabolites (phytochemicals) that exhibit a diversity of medicinal properties [8]. The majority of these compounds are used by plants as a defense mechanism against other microorganisms, herbivores, and competitors [9]. The principal phytochemicals present in plants are essential oils (EOs), phenolic compounds, alkaloids, lectins/polypeptides, and polyacetylenes [10].

EOs are a complex mixture of natural, volatile, and aromatic compounds synthesized by aromatic plants that have been often used in traditional medicine [11]. They are classified as monoterpenes and sesquiterpenes, according to the number of isoprene units, monoterpenes being the most abundant in EOs components. The second group present in EOs (less predominance) are aromatics compounds, derived from phenylpropane (mixtures of aldehydes, alcohols, phenols, methoxy derivatives, and methylenedioxy compounds) [11].

Nowadays, more than 3000 EOs have been identified and only 10% are approved for use in diverse areas (pharmaceutical, food, and cosmetic). In addition, these natural compounds are generally recognized as safe by FDA (Food and Drug Administration, US) [11–13]. A large number of biological activities have been reported for EOs such as antimicrobial, antiviral, antimycotic, antiparasitic, insecticidal, antidiabetic, antioxidant, and anticancer [14, 15]. The biological activities are related with EOs bioactive compounds, as well as the functional groups and structure arrangement from these molecules [16].

EOs exhibit antimicrobial potential against a large number of Gram-negative and Gram-positive bacteria [17, 18]. It has been observed that the mode of action of EOs is based on their ability to disrupt cell wall and cytoplasmic membrane, leading to lysis and leakage of intracellular compounds [16]. However, there is limited detailed information about how these compounds achieve this antimicrobial activity, and at the same time, additional information is required on the antimicrobial potential of pure compounds present in EOs.

**Figure 1:**
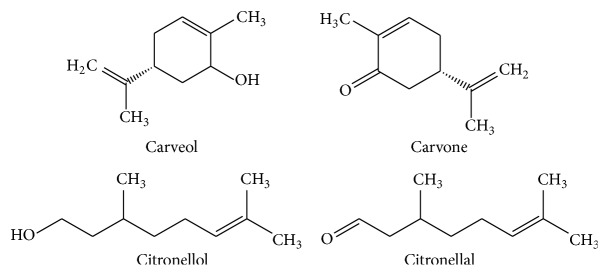
Chemical structure of selected essential oils (EOs) components.

The objectives of this work were to investigate the antibacterial activity and aspects on the mode of action of selected EOs components, carveol, carvone, citronellol, and citronellal (Figure 1), against two bacteria of clinical concern,* Escherichia coli* and* Staphylococcus aureus*.

## 2. Materials and Methods

### 2.1. Bacterial Strains and Growth Conditions

The microorganisms used in this study were* E. coli* CECT 434 and* S. aureus* CECT 976. These bacteria were previously used in antimicrobial tests with phytochemical products [19–21]. All strains were preserved at −80°C in cryovials containing liquid medium (700 *μ*L) and 30% (vol/vol) glycerol and subcultured in Mueller-Hinton agar (MHA, Merck, Germany), at 30°C during 24 h before testing.

### 2.2. Essential Oils (EOs) Components

Carveol and carvone were obtained from Sigma-Aldrich (Portugal) and citronellol and citronellal were obtained from Acros Organics (USA). Each compound (the compounds were weighed out leading to *μ*g/mL units) was tested at various concentrations in the range of 0.066 to 3000 *μ*g/mL in dimethyl sulfoxide (DMSO, 100%) (Sigma-Aldrich). The solutions of EOs components in DMSO did not exceed 10% (v/v) of the final volume of cell suspensions. Cell suspensions with DMSO (10%, v/v) and cell suspension without EOs components were used as controls. To assess the mode of action, each EOs component was tested at MIC concentration. All tests were performed in triplicate with three repeats.

### 2.3. Determination of Minimum Inhibitory Concentration (MIC) and Minimum Bactericidal Concentration (MBC)

The MIC of EOs components was determined by the microdilution broth method [22]. Briefly, after overnight growth at 30°C in Mueller-Hinton broth (MHB, Merck, Germany) an inoculum was taken and cell density OD_600 nm_ was adjusted to 0.134 ± 0.02 (1 × 10^6^ cells/mL). Afterwards, for each bacterium, at least 16 wells of sterile 96-well polystyrene microtiter plates (Orange Science, USA) were inoculated with 180 *μ*L of cells and 20 *μ*L of each compound. The percentage of DMSO did not exceed 10% (v/v) of the volume used per well (200 *μ*L). No antimicrobial activity was detected by DMSO (data not shown). The microtiter plates were then incubated for 24 h at 30°C in an orbital shaker (150 rpm). The absorbance was measured at 600 nm using a Microplate Reader (SpectraMax M2e, Molecular Devices, Inc.), and the lowest concentration of EOs components at which no growth was detected was defined as the MIC [22]. After MIC determination, 10 *μ*L of each well corresponding to the EOs components concentrations equal and above the MIC was taken and plated out on plate count agar (PCA, Oxoid, England) and incubated at 30°C for 24 h. The complete growth absence was considered as the MBC.

### 2.4. Physicochemical Characterization of Bacterial Surfaces

For the physicochemical characterization, bacterial suspension was prepared in ultrapure water at pH 6. Bacterial suspensions with each EOs component (at corresponding MIC) were incubated for 1 h at 30°C. Subsequently, physicochemical properties were determined by sessile drop contact angle measurement on bacterial lawns [23]. To determine contact angles an OCA 15 Plus (DATAPHYSICS) video based optical measuring instrument was used, allowing image acquisition and data analysis. The contact angle measurements (≤25, determination per liquid and test compounds was evaluated) were determined following the methodology described by Simões et al. [24]. The components reference values of the liquid surface tension were taken from the literature [25]. The degree of hydrophobicity was evaluated and expressed as the free energy interaction between two entities on surface immersed in water (*w*) − Δ*G*_*SWS*_ (mJ/m^2^) [26–28]. A material is considered hydrophobic if the interaction between two entities is stronger than the interaction of each separately. The surface tension components of the interacting entities can be calculated Δ*G*_*SWS*_ with the next equation:

(1) ΔGSWS=−2γSLW−γWLW+4γS+γW−+γS−γW+−γS+γS−−γW−γW+.

The Lifshitz-van der Waals components of the free surface are indicated by *γ* ^LW^, and *γ* ^+^, *γ* ^−^ represented the electron acceptor and electron donor parameters, respectively, of the Lewis acid-based component (*γ* ^AB^), with $\gamma^{AB}=2\sqrt{\gamma^{+}\gamma^{-}}$. In solid material, the surface tension components were obtained by measuring the contact angles of three different polarity liquids: *α*-bromonaphthalene (apolar), formamide (polar), and water (polar). The liquids used are known as surface tension components. The values obtained were analyzed with the next equation:

(2) $$\begin{array}{c} \left(\;1+\cos\theta \;\right)\gamma_{w}^{Tot}=2\left(\;\sqrt{\gamma_{S}^{\text{LW}}\gamma_{W}^{\text{LW}}}+\sqrt{\gamma_{S}^{+}\gamma_{W}^{-}}+\sqrt{\gamma_{S}^{-}\gamma_{W}^{+}}\;\right), \end{array}$$

where *θ* represented the contact angle and *γ* ^Tot^ = *γ* ^LW^ *γ* ^AB^.

### 2.5. Bacterial Surface Charge (Zeta Potential)

The zeta potential was determined using a Nano Zetasizer (Malvern Instrument) by applying an electric field across the bacterial suspensions [22]. The zeta potential of bacterial suspensions was measured before and after exposure to the selected EOs.

### 2.6. Assessment of Membrane Integrity: Propidium Iodide Uptake

The Live/Dead* Bac*Light kit (Invitrogen) assesses membrane integrity by selective stain exclusion [29]. This is a rapid method commonly used to determine both viable and total counts of bacteria [22]. The* Bac*Light kit is composed of two nucleic acid-binding stains: SYTO 9 and propidium iodide (PI). Bacterial strains were cultured overnight at 30°C in MHB, centrifuged (3772 g, 10 min), and washed once with saline solution (NaCl, 0.85%). Subsequently, bacteria were resuspended in NaCl to obtain an OD_600 nm_ of 0.134 ± 0.02. Then, 1 mL of each cell suspension was maintained in contact with test compounds (at corresponding MIC) for 1 h. Cell suspension with DMSO at 10% and without EOs components was used as controls. Then, bacterial suspensions were diluted (1 : 10) in NaCl and 300 *μ*L of each diluted suspension was filtered through a Nucleopore (Whatman) black polycarbonate membrane (pore size 0.22 mm) and stained with 250 mL of diluted SYTO 9 and 250 mL of diluted component PI. The dyes were left to react for 15 min in the dark, at 27 ± 3°C. The membrane was then mounted on* Bac*Light mounting oil, as described in the manufacturer's instructions. A LEICA DMLB2 microscope with mercury lamp HBO/100W/3 was used to observe stained bacteria, incorporating a color digital camera to acquire images using IM50 software (LEICA) and a ×100 oil immersion fluorescence objective. The optical filter combination for optimal viewing of stained mounts consisted in a 480–500 nm excitation filter with a 485 nm emission filter (Chroma 61000-V2 DAPI/FITC/TRITC). The total number of cells (both stains) and the number of PI stained cells (damaged) were calculated using a program path (Scan Pro 5). The total cell number and the number of PI stained cells on each membrane were estimated from minimum counts of >20 fields of view. The total cells number* per* field was approximately 50–200 cells.

### 2.7. Potassium (*K* ^*+*^) Leakage

The potassium leakage was determined using a flame emission and atomic absorption spectroscopy used for *K* ^+^ titration in solution (bacterium + EOs) [22]. The solution was filtrated after contact with the test compounds. The samples were analyzed in a GBC AAS 932 plus device using GBC Avante 1.33 software.

### 2.8. Statistical Analysis

Statistical analysis was evaluated using the NCCS, 2007 software. One way analysis of variance was performed. Differences were evaluated by using Tukey-Kramer test. The significance level in error was *P* ≤ 0.05.

## 3. Results

### 3.1. Inhibitory and Bactericidal Concentrations of EOs

The MIC and MBC values obtained for the selected EOs components against* E. coli* and* S. aureus* are shown in Table 1. All compounds presented inhibitory and bactericidal effects against* E. coli*. However, no inhibitory activity was observed against* S. aureus* with carvone, at the maximum concentration tested (3000 *μ*g/mL). Therefore, this molecule was not used against* S. aureus*, for the mode of action assays.* E. coli* was strongly inhibited by citronellol (5 *μ*g/mL), followed by carveol/carvone (200 *μ*g/mL) and citronellal (300 *μ*g/mL). For* S. aureus*, citronellol (375 *μ*g/mL) and citronellal (400 *μ*g/mL) presented the lowest MIC values followed by carveol (2000 *μ*g/mL). Regarding the bactericidal effect, citronellol and citronellal were the most effective EOs component with MBC of 15 *μ*g/mL and 500 *μ*g/mL, respectively, for* E. coli*. The MBC of both carveol and carvone against* E. coli* was 1500 *μ*g/mL. For* S. aureus*, MBC values of 400 *μ*g/mL, 800 *μ*g/mL, and 2500 *μ*g/mL were assessed for citronellol, citronellal, and carveol, respectively. Carvone showed no bactericidal activity against* S. aureus*. In general citronellol was the most effective molecule against both pathogens.

### 3.2. Effects of EOs on Bacterial Physicochemical Surface Properties: Hydrophobicity and Surface Charge

The hydrophobicity and polar and apolar components of the surface tension of* E. coli* and* S. aureus* after 1 h of exposure to selected EOs components were determined using the van Oss approach (Table 2). This method allows the assessment of the total degree of hydrophobicity of any surface in comparison with its interaction with water. Both* E. coli* and* S. aureus* have hydrophilic surfaces (Δ*G* ^TOT^ > 0 mJ/m^2^), before exposure to the EOs. In general, the application of EOs leads to changes in the physicochemical surface properties.* E. coli* cell surface (25.86 mJ/m^2^) became more hydrophilic in the presence of carveol (33.8 mJ/m^2^), carvone (33.44 mJ/m^2^), and citronellol (33.86 mJ/m^2^) and no significant effect was observed on the cell surface properties with citronellal (24.48 mJ/m^2^) (*P* > 0.05). The same behavior was verified for* S. aureus*; that is, carveol and citronellol promoted a cell surface hydrophilic character, and no alteration was found with citronellal. Moreover, the apolar properties (*γ* ^LW^) of both bacteria were not modified (*P* > 0.05) by EOs. The values of the polar component (*γ* ^AB^) of* E. coli *and* S. aureus* were reduced (*P* < 0.05) after treatment with all EOs components tested, except with citronellal for* E. coli* which acquired polar properties. The electron acceptor component (*γ* ^+^) decreased with EOs components application for both* E. coli* and* S. aureus* (except with citronellal). The values of the electron donor component increased (*P* < 0.05) after treatment with carveol (for both bacteria), carvone (for* E. coli*), and citronellol (for* E. coli*).

The zeta potential measurement provides information about the surface charge of the cells and is calculated from the mobility of cells in the presence of an electrical field under specific pH and salt concentrations.  Table 3 shows that before treatment with EOs the two bacteria tested had negative surface charge: −22.78 mV for* E. coli* and 27.10 mV for* S. aureus*. Significant changes in the cellular surface charge of* E. coli* and* S. aureus* (*P* < 0.05) were observed after exposure to the EOs (Table 3). The zeta potential values of* E. coli* became less negatives after contact with all molecules tested, wherein citronellol (−8.13 mV) promoted the greatest alteration followed by citronellal (−11.71 mV), carveol (−13.58 mV), and carvone (−18.46 mV). The same surface charge alteration patterns were observed for* S. aureus* following EOs exposure, although not so pronounced as for* E. coli*.

### 3.3. Effects of EOs Components on Bacterial Membrane Integrity

**Figure 2:**
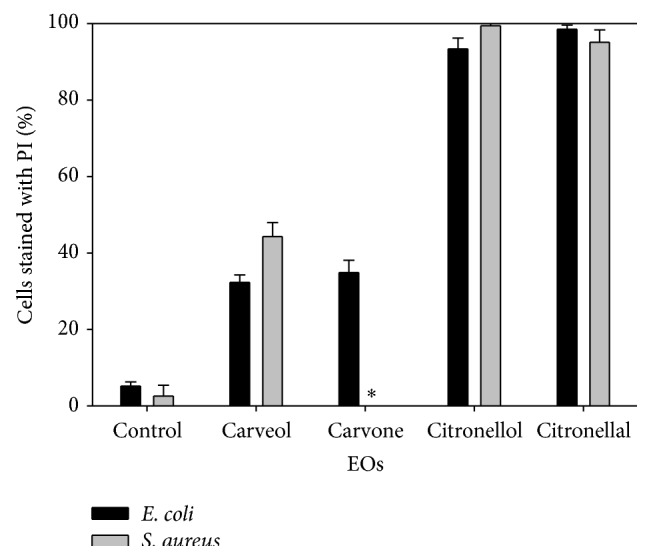
Permeability of* E. coli* and* S. aureus* to propidium iodide (PI) after 1 h of exposure to the selected EOs components at their MIC. *∗*: not evaluated. Mean values ± standard deviation for at least three replicates are illustrated.

The integrity of cell membranes can be assessed based on the ability of PI to penetrate cytoplasmic membrane. PI only penetrates cells with damaged membrane. In this way, the potential of selected EOs components to interfere with membrane integrity after 1 h exposure was analyzed (Figure 2). The PI uptake results suggest that all the tested EOs components compromise the integrity of the cytoplasmic membrane of both bacteria (*P* < 0.05). For* E. coli* the percentage of cells stained with PI after 1 h of treatment (at corresponding MIC) was carveol (32%), carvone (35%), citronellol (93%), and citronellal (98%). For* S. aureus* exposed to carveol, citronellol, and citronellal the damage in cytoplasmic membrane was about 44%, 99%, and 95% of the total cells, respectively.

### 3.4. Effects of EOs Components on Intracellular Potassium Leakage

The* K* ^*+*^ leakage determination is used to identify alterations of the cell membrane permeability. The effects of EOs components on* K* ^*+*^ release from* E. coli* and* S. aureus* cells are shown in Table 4. No loss of intracellular* K* ^*+*^ was observed for* E. coli* cells with all EOs components, at the tested concentration. For* S. aureus*,* K* ^*+*^ leakage was found with application of carveol, citronellol, and citronellal (*P* < 0.05).

## 4. Discussion

In the last decades, the incidence of human pathogens resistant to several antimicrobials has increased worldwide [7]. The lack of effectiveness of traditional antibiotics has created serious problems on the treatment of infectious diseases [30]. Therefore, it is necessary to find new alternative therapies to combat or reduce cases of infectious diseases associated with resistant pathogens. Due to the accepted safe status of some natural products, the interest in antimicrobials derived from nature has increased [10]. Indeed, a notable amount of drugs has been obtained from natural sources, particularly from plants [31]. Plants are used for thousands of years in traditional medicine to treat infections and currently they continue to play an important role in the discovery of new molecules [32]. Within plant secondary metabolites, EOs contain a diversity of bioactive compounds with chemical and structural variance that make them versatile in terms of functions. Due to their chemical variety, EOs represent a distinctive group of possible novel antimicrobial agents that have attracted special attention [33]. Although there are many reports about the antimicrobial activity of EOs from plants, few studies have been reported on the antimicrobial properties of their single molecules. In fact, most of the available studies reported the effects of eugenol, thymol, and carvacrol. In addition, information on the mode of action of individual EOs components is scarce and the specific mechanism is still not completely understood [34]. In order to contribute to the filling of these gaps, the present study provides new information on the antimicrobial activity and mode of action of four selected EOs components (carveol, carvone, citronellol, and citronellal) belonging to the class of terpenes (monoterpenoids). The MIC and MBC were assessed followed by the study of different bacterial physiological indexes (surface hydrophobicity, surface charge, PI uptake, and intracellular* K* ^+^ release).

The analysis of inhibitory and bactericidal activity showed that all EOs components tested were active at different extents against* E. coli* and* S. aureus*, with the exception of carvone to* S. aureus*. Generally, it was observed that citronellol displayed the higher inhibitory and bactericidal activity against both pathogens, followed by citronellal, carveol, and carvone. The MIC and MBC values, obtained in the present study, are in the range of those observed in others' works. In a study performed by Hussain et al. [30], citronellal demonstrated bactericidal effect against strains of* E. coli* and* S. aureus* at concentrations <1000 *μ*g/mL and <490 *μ*g/mL, respectively. Cimanga et al. [35] tested the antibacterial activity of EOs from 15 Congolese aromatic plant species against 11 clinical bacterial isolates, including* E. coli* and* S. aureus* strains. They verified that EOs of* Eucalyptus citriodora*, in which 73% of their constituents are citronellal and 6% are citronellol, presented higher antimicrobial activity against* E. coli* strains (isolated from feces and urine) than* S. aureus *strains (isolated from abscess). Contrarily, Wattanastcha et al. [36] observed that citronellal was effective on growth inhibition of* S. aureus*; however, for* E. coli* no antimicrobial activity was observed. Moreover, Griffin et al. [37] found that citronellol and citronellal exhibited lower antimicrobial activity against* E. coli *compared with* S. aureus* while carveol and carvone showed an opposite behavior on these pathogens. Simic et al. [38] obtained an MIC of 6 *μ*g/mL and 2 *μ*g/mL with* Cymbopogon winterianus* (citronellal and citronellol as main EOs components) and* Carum carvi* (carvone as main EOs component) EOs, respectively, against* E. coli*. This effect was more evident against* S. aureus* with both EOs (*C. winterianus*: 2 *μ*g/mL;* C. carvi*: 2 *μ*g/mL). Similar behavior was found relative to MBC. EO of* C. carvi*, where carvone is the dominant constituent, has stronger antibacterial activity than* C. winterianus* EOs. In the present study, Gram-positive bacterium was more resistant than the Gram-negative one to the EOs components tested. This behavior was similar to that obtained by other authors [21, 22, 39, 40]. In this sense, some studies suggest that the difference in the susceptibility to antimicrobials between Gram-positive and Gram-negative bacteria could be attributed to the cell envelope (cytoplasmic membrane and/or outer membrane and cell wall) structure and composition [41, 42]. In Gram-negative bacteria cell wall is more complex. It is constituted by a thin peptidoglycan layer adjacent to cytoplasmic membrane and an outer membrane (OM) composed by phospholipids and lipopolysaccharides (LPS) [43]. The passage through the OM is regulated by the presence of hydrophilic channels, named porins, which generally exclude the entry of hydrophobic substances. In this study the presence of an OM, in addition to the cytoplasmic membrane in Gram-negative bacteria, did not increase the resistance to EOs components. In fact, some compounds have now demonstrated their capacity to disrupt the OM through the release of LPS [44]. Additionally, certain EOs phenolic constituents (carvacrol and thymol) with hydrophobic character have revealed potential to interact with OM with consequent bactericidal activity [45, 46]. On the other hand Gram-positive bacteria lack the OM, but the cell wall is formed by a thicker peptidoglycan layer that confers rigidity to cells and makes it difficult to penetrate by antimicrobials. This characteristic can be one explanation to the lower activity of EOs constituents against the Gram-positive bacterium* S. aureus*.

There are several targets described for EOs, namely, destabilization of bacterial membranes, membrane proteins damage, proton motive force depletion, and cell contents release [47–52]. In most of the cases EOs confer antimicrobial activity by damaging the cell wall and membranes, which lead to cell lysis and leakage of cell contents [16]. Several reports suggest that the antimicrobial mode of action of EOs and corresponding components depends on their chemical composition and on the amount of single components. Also, the presence and location of functional groups in the molecule can affect its bioactivity [43]. In this study the acyclic compounds presented higher antimicrobial activity compared with cyclic compounds. Concerning the acyclic compounds, the difference between citronellol and citronellal is the substitution of an alcohol by an aldehyde group. This replacement and consequently the absence of a double bond in citronellol were therefore responsible for their higher activity against both bacteria. The presence of a carbonyl group in carvone instead of a hydroxyl group in position 3 of carveol appears to have been responsible for the lack of activity of carvone against* S. aureus*. However, this difference did not affect the activity against* E. coli*.

Regarding the physicochemical surface properties of bacterial cells, in general the selected EOs components changed the hydrophobicity and also the surface tension parameters of both bacteria. It is known that EOs have a hydrophobic nature, which allows them to penetrate microbial cells and cause disruption of the cell wall/membranes structure and impairment of cell functions. This leads to an increase in the permeability due to the incapability to separate the EOs from bacterial cell membranes [43]. Thus, it is possible to hypothesize that the hydrophobicity changes of bacterial membranes, after treatment with EOs constituents, can lead to destabilization of the phospholipid bilayer of cytoplasmic membrane of Gram-positive bacteria. In the same way, these compounds may also have affected the hydrophobic character of LPS from the OM, in addition to the interaction with the cytoplasmic membrane. As cell membranes provide a barrier that is indispensable for many cellular processes taking place within the cells, their damage entails deleterious effects that can cause cell inactivation and/or death.

Another characteristic that plays a vital role in the microbial balance and resistance to antimicrobials is the charge of the cell surfaces [22]. Normally, at physiological conditions bacterial cells have a negative surface charge due to the presence of anionic groups (e.g., carboxyl and phosphate) in the membrane [53–55]. Nonetheless, the magnitude of the charge varies with the species and can be affected by several conditions, including the age of the culture, ionic strength, and pH [55, 56]. In this study, zeta potential measurements demonstrated that after exposure to EOs components a reduction in the negative surface charge (less negative values) of* E. coli* and* S. aureus* was observed. This change in the surface charge was more evident for the Gram-negative bacterium. This behavior suggests that EOs components interacted more strongly with* E. coli* than with* S. aureus*. Similar findings were obtained by Kim et al. [57] with eight EOs constituents (citral, carvacrol, geraniol, terpineol, perillaldehyde, eugenol, linalool, and citronellal) against four Gram-negative bacteria (*E. coli*,* E. coli *0157:H7,* Salmonella typhimurium,* and* Vibrio vulnificus*) and one Gram-positive bacterium (*Listeria monocytogenes*). The majority of studies indicate that in general EOs and their components are most effective against Gram-positive bacteria, because Gram-negative bacteria do not allow the entrance of hydrophobic molecules [43]. However, this study shows that the presence of an OM was not relevant for antimicrobial resistance. Moreover, the cell shape may be involved in the distinct susceptibility of* E. coli* and* S. aureus*. Normally, rod shaped bacterial cells are more sensitive to EOs than coccoid cells [58].

Cytoplasmic membrane permeabilization was observed based on the uptake of PI, a nucleic acid stain to which intact cell membrane is usually impermeable. The percentage of* S. aureus* cells with damaged membrane after exposure to carveol and citronellol was higher than for* E. coli*. Citronellol and citronellal were the most effective molecules against* S. aureus* and* E. coli*, respectively. In the presence of these molecules the percentage of damaged cells reached almost 100% of the total population. Based on these results, it is possible to conclude that membrane/cell wall permeabilization can be related with alterations on their physicochemical properties (hydrophobicity and charge). Indeed, Sikkema et al. [59, 60] showed that as a result of lipophilic character of monoterpenoid components they are preferentially partitioned from an aqueous phase into membrane structures. This results in membrane expansion, increased membrane fluidity and permeability, disturbance of membrane-embedded proteins, inhibition of respiration, and alteration of ion transport processes [61]. Helander and coworkers [45] described the effect of various EOs components on the OM permeability in Gram-negative bacteria, evidencing that monoterpene capture is determined by the permeability of the envelope of the target microorganism.

The cytoplasmic membrane has a very important role in the maintenance of cellular homeostasis, as it controls the input and output of intracellular components. The internal environment of cells is generally rich in* K* ^*+*^, so their presence in the extracellular medium is an indication of serious and irreversible cytoplasmic membrane damage [31, 62]. Some EOs are recognized to have membrane active properties against several microorganisms, causing leakage of cell constituents, including* K* ^*+*^ [49, 63–65]. Cox and collaborators [63] evaluated the antimicrobial mode of action of tea tree oil against* E. coli* and* S. aureus* strains and reported increased uptake of PI and leakage of* K* ^*+*^. In this study* K* ^*+*^ release was verified for* S. aureus* treated with carveol, citronellol, and citronellal. Nevertheless, no* K* ^*+*^ release was found for* E. coli* with all molecules. This suggests that selected EOs components were only able to cause structural alterations of the outer envelope without promoting release of cellular content. However, these cell surface changes were sufficient to induce cell death. Moreover, although EOs constituents did not cause* K* ^*+*^ leakage, they promoted PI uptake. Therefore, it can be hypothesized that the* K* ^*+*^ absence in the supernatant was due to retention in the thin layer of* E. coli* cell wall. In fact, given the increased permeability to PI, it seems unlikely that the cytoplasmic membrane has remained impermeable to* K* ^*+*^.

In conclusion, the selected EOs components have an interesting antimicrobial activity (except carvone against* S. aureus*), targeting mainly the bacterial membranes. Based on the overall results, it can be hypothesized that these molecules interact strongly with cell surface constituents such as membrane proteins and other molecules essential for the microbial growth and survival. After binding to the cell surface, they can form a monolayer around the cell that modifies the electrostatic potential and hydrophobicity and therefore destabilizes the membrane integrity, resulting in internal cellular components release. Also, the results propose that the selected EOs components can be a natural-based alternative to conventional synthetic antimicrobials to control bacterial infections caused by* E. coli* and* S. aureus*, particularly topical infections. However, more studies are necessary to explore their toxicity to mammalian cells and drug-like properties (pharmacokinetic and pharmacodynamic) in order to ascertain their potential as therapeutic agents, including for the treatment of systemic infections.

## Figures and Tables

**Table 1:** MIC and MBC of selected EOs components for *E. coli* and *S. aureus*.

EOs	MIC (*μ*g/mL)		MBC (*μ*g/mL)	
	*E. coli *	*S. aureus *	*E. coli *	*S. aureus *
Carveol	200	2000	1500	2500
Carvone	200	NA	1500	NA
Citronellol	5	375	15	400
Citronellal	300	400	500	800

NA: no activity (MIC/MBC > 3000 *μ*g/mL).

**Table 2:** Hydrophobicity (Δ*G* ^TOT^) and apolar (*γ* ^LW^) and polar (*γ* ^AB^) components of the surface tension of *E. coli* and *S. aureus* after 1 h of exposure to MIC of selected EOs components.

Bacteria	EOs	Surface tension parameters				
		*γ* ^LW^	*γ* ^AB^	*γ* ^+^	*γ* ^−^	Δ*G* ^TOT^ (mJ/m^2^)
*E. coli *	Control	33.58 ± 1.99^b^	20.74 ± 1.98^bc^	2.17 ± 0.42^bc^	49.52 ± 0.03^a^	25.86 ± 0.31^a^
	Carveol	34.40 ± 0.74^b^	16.71 ± 1.94^a^	1.28 ± 0.29^a^	54.60 ± 0.62^b^	33.80 ± 0.76^b^
	Carvone	34.45 ± 0.38^b^	17.33 ± 0.30^ab^	1.37 ± 0.03^ab^	54.64 ± 0.51^b^	33.44 ± 0.24^b^
	Citronellol	35.88 ± 0.90^b^	15.74 ± 1.17^a^	1.13 ± 0.17^a^	54.66 ± 0.17^b^	33.86 ± 0.48^b^
	Citronellal	27.8 ± 0.95^a^	25.93 ± 1.59^c^	3.41 ± 0.43^c^	49.34 ± 0.42^a^	24.48 ± 0.97^a^

*S. aureus *	Control	35.44 ± 1.15^a^	17.09 ± 0.28^a^	1.48 ± 0.05^a^	49.57 ± 0.91^a^	27.68 ± 1.35^a^
	Carveol	34.14 ± 0.60^a^	15.74 ± 2.00^a^	1.10 ± 0.32^a^	56.82 ± 2.49^b^	37.11 ± 3.96^b^
	Citronellol	35.05 ± 0.38^a^	14.87 ± 0.34^a^	1.10 ± 0.03^a^	50.03 ± 0.56^a^	29.23 ± 0.64^b^
	Citronellal	34.15 ± 0.01^a^	16.74 ± 0.35^a^	1.47 ± 0.11^a^	47.55 ± 1.53^a^	25.56 ± 2.04^a^

^a–c^Different superscripts within the same column indicate statistical significant differences (*P* < 0.05). Mean values ± standard deviation for at least three replicates are illustrated.

**Table 3:** Zeta potential values (mV) of *E. coli* and *S. aureus* after 1 h of exposure to MIC of selected EOs components.

EOs	Zeta potential (mV)	
	*E. coli *	*S. aureus *
Control	−22.78 ± 2.85^a^	−27.10 ± 2.42^a^
Carveol	−13.58 ± 1.02^c^	−18.86 ± 2.02^b^
Carvone	−18.46 ± 1.37^b^	NE
Citronellol	−8.13 ± 1.21^d^	−16.03 ± 0.66^b^
Citronellal	−11.71 ± 0.73^c^	−15.16 ± 1.00^b^

^a–d^Different superscripts within the same column indicate statistical significant differences (*P* < 0.05). NE: not evaluated. Mean values ± standard deviation for at least three replicates are illustrated.

**Table 4:** Concentration of *K* ^+^ (*μ*g/mL) in solution of *E. coli* and *S. aureus* after 1 h of exposure to MIC of selected EOs components.

EOs	*E. coli *	*S. aureus *
Control	0.0 ± 0.0	0.0 ± 0.0^a^
Carveol	0.0 ± 0.0	0.34 ± 0.098^b^
Carvone	0.0 ± 0.0	NE
Citronellol	0.0 ± 0.0	0.63 ± 0.008^c^
Citronellal	0.0 ± 0.0	0.48 ± 0.053^bc^

^a–d^Different superscripts within the same column indicate statistical significant differences (*P* < 0.05). NE: not evaluated. Mean values ± standard deviation for at least three replicates are illustrated.
